# Studies on the contribution of PPAR Gamma to tuberculosis physiopathology

**DOI:** 10.3389/fcimb.2023.1067464

**Published:** 2023-04-28

**Authors:** Ariana Díaz, Luciano D’Attilio, Federico Penas, Bettina Bongiovanni, Estefanía Massa, Agata Cevey, Natalia Santucci, Oscar Bottasso, Nora Goren, María Luisa Bay

**Affiliations:** ^1^ Facultad de Ciencias Médicas, Instituto de Inmunología Clínica y Experimental de Rosario (IDICER), CONICET - Universidad Nacional de Rosario, Rosario, Argentina; ^2^ Facultad de Medicina, Instituto de Investigaciones Biomédicas en Retrovirus y SIDA (INBIRS), CONICET - Universidad de Buenos Aires, Buenos Aires, Argentina; ^3^ Facultad de Ciencias Bioquímicas y Farmacéuticas, Universidad Nacional de Rosario, Rosario, Argentina

**Keywords:** tuberculosis, cortisol, DHEA, PPARγ, infectious disease

## Abstract

**Introduction:**

Tuberculosis (TB) is a major health problem characterized by an immuno-endocrine imbalance: elevated plasma levels of cortisol and pro- and anti-inflammatory mediators, as well as reduced levels of dehydroepiandrosterone. The etiological agent, Mycobacterium tuberculosis (Mtb), is captured by pulmonary macrophages (Mf), whose activation is necessary to cope with the control of Mtb, however, excessive activation of the inflammatory response also leads to tissue damage. Glucocorticoids (GC) are critical elements to counteract the immunoinflammatory reaction, and peroxisome proliferator-activated receptors (PPARs) are also involved in this regard. The primary forms of these receptors are PPARϒ, PPARα, and PPARβ/δ, the former being the most involved in anti-inflammatory responses. In this work, we seek to gain some insight into the contribution of PPARϒ in immuno-endocrine-metabolic interactions by focusing on clinical studies in pulmonary TB patients and in vitro experiments on a Mf cell line.

**Methods and results:**

We found that TB patients, at the time of diagnosis, showed increased expression of the PPARϒ transcript in their peripheral blood mononuclear cells, positively associated with circulating cortisol and related to disease severity. Given this background, we investigated the expression of PPARϒ (RT-qPCR) in radiation-killed Mtb-stimulated human Mf. The Mtb stimulation of Mf derived from the human line THP1 significantly increased the expression of PPARϒ, while the activation of this receptor by a specific agonist decreased the expression of pro- and anti-inflammatory cytokines (IL-1β and IL-10). As expected, the addition of GC to stimulated cultures reduced IL-1β production, while cortisol treatment together with the PPARϒ agonist lowered the levels of this proinflammatory cytokine in stimulated cultures. The addition of RU486, a glucocorticoid receptor antagonist, only reversed the inhibition produced by the addition of GC.

**Conclusion:**

The current results provide a stimulating background for further analysis of the interconnection between PPARs and steroid hormones in the context of Mtb infection.

## 1 Introduction

Tuberculosis (TB) is one of the top ten causes of death worldwide. Until the coronavirus (COVID-19) pandemic, TB was the leading cause of death attributed to a single infectious agent, ranking above HIV/AIDS (Global Tuberculosis Report, 2022). According to WHO estimates, there were 10.6 million TB cases in 2021, reaching 1.4 million deaths, although these numbers are considered underestimates as the COVID-19 pandemic delayed global TB control services, reversing years of progress made in the fight against this disease (No Author, 2019).

TB is an ancient chronic infectious disease caused by the bacillus *Mycobacterium tuberculosis* (Mtb), which is spread when sick people expel bacteria into the air, for example, by coughing. The disease usually affects the lungs (pulmonary TB) but can also affect other body sites (extrapulmonary TB). Commonly, the host immune response (IR) controls Mtb replication, by promoting mycobacterial clearance or leading to the establishment of a latent infection, which ultimately depends on a fine balance between the pathogen and the specific IR (Ernst, 2012).

Mtb mainly infects macrophages (Mf) which need to be activated by the cellular IR for containing mycobacteria (Th1 pattern activation) (Russell, 2001; Hestvik et al., 2005; Jordao et al., 2008). Cytokines such as IFN-γ, secreted by T lymphocytes, and TNF-α are essential to achieve classical macrophage activation (M1) and intracellular pathogen elimination (Kaufmann, 2001; O’Garra et al., 2013). At the same time, an exacerbated IR and the ensuing excessive secretion of inflammatory mediators turn out to be detrimental to host tissues through immunopathological processes, which is quite pathognomonic of diseases of chronic nature like TB. In line with this, newly diagnosed TB patients (T0) show a marked consumption state together with a lower body mass index (BMI), in addition to an immune-endocrine-metabolic (IEM) imbalance (Santucci et al., 2011; Bottasso et al., 2013; Díaz et al., 2017). This mainly consists of increased circulating amounts of pro- and anti-inflammatory cytokines together with increased cortisol and lowered dehydroepiandrosterone (DHEA) values, partly associated with the degree of pulmonary involvement (Bottasso et al., 2013; Díaz et al., 2017; D’Attilio et al., 2018).

Within the broad array of regulatory processes which are likely to take place during Mtb infection, the contribution of the peroxisome proliferator-activated receptors (PPARs), which belong to the nuclear receptor superfamily, is worth exploring. PPARs are a family of 3 ligand-activated transcription factors: PPARα (NR1C1), PPARβ/δ NR1C2), and PPARγ (NR1C3). These PPARs are encoded by different genes but show similar structural features, which include an amino-terminal modulatory domain, a DNA binding domain, and a carboxyl-terminal ligand binding domain. All PPARs act as heterodimers with the retinoid X receptor (RXRs) and play important roles in the regulation of metabolic pathways, including those of lipid biosynthesis and glucose metabolism, as well as in a variety of cell functions: differentiation, proliferation, apoptosis pathways and inflammation (Blitek and Szymanska, 2017; Portius et al., 2017; Christofides et al., 2021).

PPAR ligands, in particular those of PPARα and PPARγ, inhibit the activation of inflammatory gene expression and can negatively interfere with proinflammatory transcription factor signaling pathways in vascular and inflammatory cells. Furthermore, PPAR levels are differentially regulated in a variety of inflammatory disorders in men, for which PPAR ligands may constitute promising therapies (Moraes et al., 2006). Evidence indicates that chronic metabolic diseases accompanied by immunological-endocrine disturbances, i.e., diabetes and metabolic syndrome, present a higher risk of infections together with a lower bactericidal capacity from the innate immune cells. In this regard, treatment with thiazolidinedione drugs like pioglitazone (synthetic PPARγ ligand), not only diminished the inflammation but also increased the capability of host cells to cope with pathogens, in addition to modulating hormone production and glucolipid homeostasis (Sharma et al., 2017). A study in a murine model of sepsis showed that treatment with the agonist pioglitazone improved mouse survival by enhancing the neutrophil bacterial clearance and promotion of an anti-inflammatory milieu at the site of infection (Ferreira et al., 2014). In the case of TB, some studies showed that mycobacterial infections coexist with an increased expression and activation of PPARγ at the Mf level which leads to changes in intracellular lipid homeostasis (foamy Mf) and its activation profile, promoting an M2 pattern more favorable to Mtb survival (Almeida et al., 2012).

Given this background and considering the substantial role of IEM disturbances in TB pathophysiology, we sought to investigate the relationship of PPARγ with some IEM components in the setting of clinical pulmonary TB, at the time of diagnosis and throughout the course of specific treatment to get a better understanding on their implication in the processes dealing with disease development and resolution. We also carried out *in vitro* experiments in a macrophage cell line for a more in deep analysis of the reciprocal influences between PPARγ and glucocorticoids.

## 2 Materials and methods

### 2.1 Subjects

Thirty-nine adults who were diagnosed with lung TB based on clinical and radiological findings and identification of TB bacilli in sputum were enrolled and followed for up to nine months in a prospective cohort study of TB treatment. This observational cohort included patients with neither HIV coinfection nor multidrug-resistant TB. Patients had mild (n = 9), moderate (n = 15), or advanced (n = 15) disease according to the radiological findings, corresponding to previously described criteria (Mahuad et al., 2004).

Antituberculosis therapy consisted of six months of rifampicin and isoniazid, initially supplemented by two months of pyrazinamide and ethambutol. Among the 39 recruited patients, blood samples at all time points (see 3.1.1.1 Sample Collection) were available in 24 of them (mild = 2, moderate = 13, and severe = 9). Age-matched healthy controls (HCo, n = 24) living in the same area and without known contact with TB patients, were incorporated as controls. Exclusion criteria for all participants included pathologies affecting the hypothalamus-pituitary-thyroid or gonadal-axis, or direct compromise of the adrenal gland, pregnancy, contraceptive drugs, age under 18, or systemic or localized pathologies requiring treatment with corticosteroids or immunosuppressants. The study protocol was approved by the Ethical Committee of the Faculty of Medical Sciences, National University of Rosario (Resolution n° 6625/2018), and the Centenario Hospital of Rosario (Resolution n° 528). The study was conducted following the 1964 Helsinki declaration and its later amendments. All volunteers gave their written consent before participating in the study.

#### 2.1.1 Sample collection

Blood samples were obtained from TB patients at the time of diagnosis (before initiation of the treatment, T0) and 2, 4, and 6 months (T2, T4, and T6) after starting the specific antituberculosis treatment (Global tuberculosis report, 2021). Also, an additional sample was obtained three months after the end of treatment completion (T9). All samples were taken between 8:00 and 9:00 a.m. with and without EDTA and then centrifuged. Aprotinin (100U/mL; Aprotinin from bovine lung, Sigma) was added to the plasma shortly after collection and the samples were preserved at −80^°^C. One blood sample was obtained from age- and sex-matched HCo and processed in the same way (Mahuad et al., 2004; Díaz et al., 2017).

#### 2.1.2 Mononuclear cell isolation

After blood centrifugation, the buffy coat was separated and diluted 1:1 in RPMI 1640 (Invitrogen) containing standard concentrations of L-glutamine, penicillin, and streptomycin (culture medium). The cell suspension was layered over a Ficoll-Paque (density 1.077, Amersham Biosciences) and centrifuged at 400 g for 30 min. From this column, PBMCs were obtained.

#### 2.1.3 Flow cytometry

A sample of EDTA anticoagulated whole blood was incubated with BD Tritest CD4FITC/CD8PE/CD3PerCP reagent, another with CD3FITC/CD19PE, and the third one with CD45FITC/CD14PE (all reagents of BD Biosciences) and isotype controls, according to the manufacturer’s instructions (Díaz et al., 2015). Stained cells were analyzed with a FACSAria II flow cytometer (BD Biosciences). The percentage of positive cells and the mean fluorescence intensity (arbitrary units) for a specific marker were calculated using FACSDiva software (BD Biosciences). For each sample, 30.000 events were recorded.

#### 2.1.4 Evaluation of immunological mediators and hormones

Cytokine and hormone levels in plasma were measured by using commercial ELISA kits (BD Biosciences, IFN-γ, IL-6, detection limit -DL-: 4.7 and 2 pg/ml, respectively), or EIA assays (DRG Systems, DL: 2.5 and 0.108 ng/ml, for cortisol and DHEA, respectively).

#### 2.1.5 RNA isolation, cDNA synthesis, and RT-qPCR

Total RNA was isolated from PBMCs using TRIzol (Invitrogen). RNA pellets were dissolved in Diethyl pyrocarbonate (DEPC) sterile water and stored at –80°C. RNA quantity and integrity were assessed as performed earlier (D’Attlio et al., 2011). cDNA was synthesized from 2 μg of total RNA by extension of oligo dT primers (Invitrogen) with M-MuLV reverse transcriptase (Thermo Fisher Scientific) in a final volume of 40 μl DEPC sterile water. cDNA was stored at –80°C until use. RT-qPCR was performed with the StepOnePlus (96-well) Real-Time PCR Systems (Applied Biosystems) using 3 μl of cDNA dilution, 0.4 μM of each primer, and 3 μl of 5x HOT FIREPol EvaGreen qPCR Mix Plus (Rox) (Solis BioDyne), the final volume of 15 μl. Thermal cycling conditions were as follows: 10 min at 95°C followed by 45 PCR cycles of denaturing at 95°C for 20 s, 30 s for annealing at 60°C, and 20 s for elongation at 72°C. Fluorescence readings were performed for 10 s at 80°C before each elongation step. To normalize the expression of every gene, the transcript of PPIA [peptidylprolyl isomerase A (Cyclophilin A)] was used as an endogenous control in each mononuclear cell sample (He et al., 2008). Serially diluted cDNA samples were used as relative external standards in each run, to make “The Relative Standard Curve Method” for the relative quantification of gene expression, as performed formerly (D’Attlio et al., 2011). Similarity and homogeneity of PCR products from samples were confirmed by automated melting curve analysis (StepOne Software, Applied Biosystems), which revealed the melting temperature values of the PCR products. Selected primers are detailed in **Table 1**. Data were expressed as fold change of the relative expression levels of the gene of interest normalized by the relative expression levels of PPIA.

**Table 1:** RT-qPCR nucleotide primer sequence.

Transcript	Forward primer	Reverse primer	Size
CycA	CycA-F	CycA-R	101 bp
PPIA GeneID: 5478	5’-ggt cct ggc atc ttg tcc at-3’	5’-ttg ctg gtc ttg cca ttc ct-3’	
PPARγ	PPARγ-F	PPARγ-R	
NR1C3, GeneID: 5468	5’-ttt cag aaa tgc ctt gca gtg g-3’	5’-ctt tcc tgt caa gat cgc cct c-3’	222 bp
PPARα	PPARα-F	PPARα-R	
NR1C1, GeneID: 5465	5’-cct ttt tgt ggc tgc tat c-3’	5’-gtg gag tct gag CAC at t-3’	106 bp

### 2.2 Cell preparations

The characteristics of THP-1, a human monocytic leukemia cell line, have been described previously in detail (Tsuchiya et al., 1980). This cell line was grown in suspension cultures in Tissue Culture Medium RPMI-1640 supplemented with 10% of heat-inactivated fetal bovine serum and antibiotic (Penicillin-Streptomycin, Gibco, Invitrogen) at 37°C in 5% CO_2_. THP-1 cells were cultured in complete RPMI-1640 containing 30 ng/ml phorbol-12-myristate-13-acetate (PMA, Sigma) and plated for differentiation to macrophages. Twenty-four hours later supernatants were removed and complete RPMI-1640 was added for 48 h before the stimulation.

#### 2.2.1 Stimulation of macrophages with *M. tuberculosis* and treatment with cortisol and/or PPARγ agonist

Macrophages were cultured in quadruplicate in flat-bottomed 24-well dishes (4 × 10^5^ cells/well in 0.6 mL) in RPMI 1640 (Gibco, Invitrogen) with 10% of heat-inactivated fetal bovine serum (Gibco, Invitrogen) and antibiotic (Penicillin-Streptomycin, Gibco, Invitrogen) and cultured for 24 h at 37°C in 5% CO_2_, with or without the addition of *M. tuberculosis* strain H37Rv gamma irradiated (Mtbi; 8 µg/mL, Colorado University, USA), 15-Deoxy-Δ^12,14^-prostaglandin J_2_ -15dPGJ_2_- a PPARγ natural ligand (2 μM, Sigma) and/or physiological concentrations of cortisol (10^-6^ M, Sigma) (Mahuad et al., 2004). Stock 15dPGJ2 and cortisol solutions were prepared in ethanol. Thereafter, stock solutions were diluted in a culture medium to final concentrations. Treatment with 15dPGJ2 was performed 30 min before the addition of Mtbi and/or cortisol. In some cultures, RU486 (RU486 or Mifepristone 1 μM, Sigma-Aldrich), a cortisol receptor antagonist, was used 5 min before the addition of the hormone. Supernatants were collected to assess cytokine production and cells were preserved in TRIzol (Invitrogen) for mRNA extraction.

### 2.3 Statistical analysis

Statistical comparisons were performed by the Mann-Whitney U and Kruskal-Wallis followed by *post hoc* comparisons when applicable since some variables under analysis deviated from a normal distribution. Paired comparisons during treatment were done by the Friedman analysis of variance. Associations between variables were analyzed using the Spearman correlation test. A value of p< 0.05 was regarded as statistically significant.

## 3 Results

### 3.1 Features of study groups

There was no sex- or age-related differences and the frequency of Bacillus Calmette–Guerin vaccination between study groups, although the body mass index (BMI, weight/height^2^) was significantly decreased in TB patients (**Table 2**). Newly diagnosed patients did not differ from controls in terms of erythrocyte counts (**Table 3**) but showed an increase in the leukocyte numbers (p<0.05) with a high percentage of neutrophils and low values of lymphocytes (p<0.05). Consequently, the ratio between the percentage of neutrophils and lymphocytes (NLR) was increased (**Table 3**), compatible with systemic inflammation (Song et al., 2021; Buonacera et al., 2022). From the second month of anti-TB treatment, these hematological variables reached values similar to those of the HCo. Eosinophils were found to increase during the specific treatment and remained high even after its termination (**Table 3**). With regards to erythrocyte sedimentation rate (ESR), TB cases displayed a significant increase at T0 and T2, further decreasing to values seen in HCo (**Table 3**). Levels of liver enzymes among treated patients remained within normal values (data not shown).

**Table 2:** Main features of study groups.

	Study groups		
Parameters	HCo (n=26)	TB (n=24)	p
**Age**	50.5 (25.8 - 57.3)	45.0 (21.5 - 54.8)	ns
**Sex (M/F)**	24/2	22/2	ns
**BMI**	27.4 (25.1 - 30.8)	19.9 (18.3 - 23.8)	p<0.001
**BCG (%)**	95%	80%	ns

Data are represented as median and interquartile ranges. BMI, body mass index (weight/height^2^); BCG, Bacillus Calmette–Guerin vaccination; HCo, healthy controls; TB, patients with pulmonary tuberculosis; ns, not significant.

**Table 3:** Quantification of erythrocytes, hemoglobin, percentage of hematocrit, leukocytes, leukocyte formula, platelets, erythrocyte sedimentation rate (ESR), and the ratio between the percentage of neutrophils and lymphocytes (NLR) in patients with TB and healthy controls.

Parameters	Study groups					
	HCo (n=26)	TB (n=24)				
		T0	T2	T4	T6	T9
**Red blood cells**	5.1	4.9	5.1	5,0	5,0	5.1
**(106/µl) [rv: 3.5-5.5]**	(4.9-5.2)	(4.5-5.6)	(4.9-5.6)	(4.7-5.3)	(4.6-5.3)	(4.7-5.5)
**HGB (g/dl)**	14.8	13.7 *	14.6 #	14.5 #	14.7	15.1 #
**[rv: 11-16]**	(14.3-15.5)	(11.9-14.5)	(13.8-15.3)	(12.9-15.1)	(13.5-15.3)	(14.1-15.5)
**Hematocrit (%)**	44.1	41.7 *	44.0 #	43.1	43.5	44.5 #
**[rv: 35-50]**	(42.3-44.9)	(37.5-44.8)	(41.8-45.8)	(39.7-44.9)	(40.5-45.9)	(41.6-47.1)
**White blood cells**	7.2	7.9 *	5.7 #	5.6 #	7.4 #	7.2 #
**(103/µl) [rv: 4-9]**	(5.4-8.1)	(6.8-10.6)	(4.8-7.2)	(4.8-8.9)	(5.2-8.0)	(5.6-8.9)
**Neutrophils (%)**	57.8	71.1 *	57.5 #	53.0 #	58.6 #	58.7 #
**[rv: 45-65]**	(52.0-63.3)	(56.0-73.9)	(51.5-62.7)	(44.9-61.2)	(46.3-60.6)	(47.1-62.9)
**Eosinophils (%)**	2.6	2,0	3.2 #	4.0 *#	4.0 *#	4.0 *#
**[rv: 0-4]**	(2.0-3.5)	(0.7-3.5)	(2.3-6.5)	(2.4-5.7)	(2.2-5.4)	(2.7-6.3)
**Basophils (%)**	0.8	0.5	0.8	0.9	0.9	1,0
**[rv: 0-1]**	(0.4-1.0)	(0.0-1.0)	(0.6-1.4)	(0.4-1.3)	(0.5-1.4)	(0.7-1.3)
**Lymphocytes (%)**	28.1	16.5 *	25.6 #	30.1 #	27.4 #	25.6 #
**[rv: 25-35]**	(25.0-35.2)	(13.7-25.3)	(19.2-33.0)	(24.7-37.4)	(23.3-34.8)	(22.6-34.8)
**Monocytes (%)**	8.1	9.8	9.5	9.1	9.4	9.5
**[rv: 0-12]**	(6.8-10.0)	(7.2-13.0)	(7.4-11.6)	(8.1-12.6)	(6.9-11.2)	(8.2-10.5)
**Platelets (103/µl)**	236.5	323.0 *	253.0 #	220.5 #	215.0 #	248.0 #
**[rv: 150-400]**	(207.8-289.5)	(259.0-417.0)	(224.0-352.0)	(202.5-307.5)	(190.5-277.0)	(199.0-275.0)
**ERS (mm/1°h)**	5,0	44.0 *	11.0 *#	5.0 #	6.0 #	6.0 #
**[rv: 1-15]**	(2.0-11.0)	(17.3-75.8)	(4.5-21.5)	(4.0-18.0)	(3.0-13.0)	(2.8-10.5)
**Neutrophils/lymphocytes**	2.1	4.5 *	2.2 #	1.6 #	2.1 #	2.4 #
**ratio (NLR)**	(1.4 - 2.6)	(2.1 - 5.2)	(1.5 - 3.2)	(1.2 - 2.5)	(1.2 - 2.6)	(1.3 - 2.7)

Data are represented as median and interquartile ranges. HCo, healthy controls; TB, patients with pulmonary tuberculosis; T0, time at diagnosis; T2, T4, and T6, 2, 4, and 6 months following the initiation of anti-bacillary treatment; T9, 3 months following treatment completion. HGB, hemoglobin; ESR, erythrocyte sedimentation rate; NLR, the ratio between the percentage of neutrophils and lymphocytes; rv, reference values. *p<0.05 vs. HCo; #p<0.05 vs. T0.

**Figure 1:**
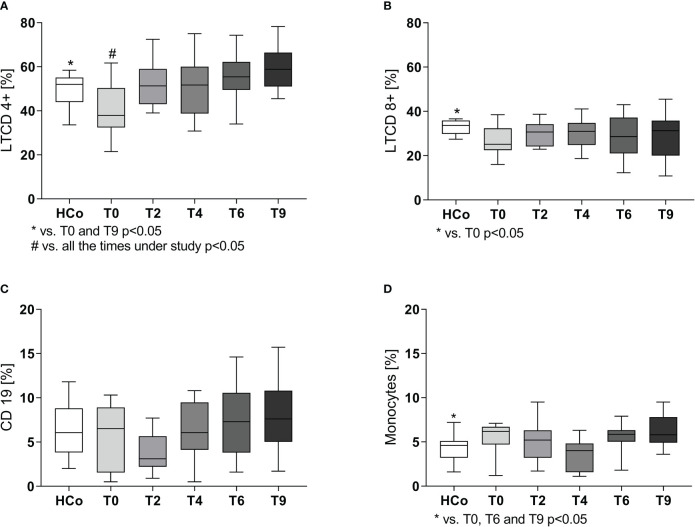
CD4+ **(A)**, CD8+ **(B)**, B **(C)** lymphocytes, and monocytes **(D)** populations in TB patients during specific treatment. Boxes represent the median (line) and interquartile range, with minimal and maximum values. HCo (n = 24): healthy controls; T0: time at diagnosis; T2, T4, and T6: 2, 4, and 6 months following the initiation of anti-bacillary treatment; T9: 3 months following treatment completion. Twenty-four TB patients were studied at all time points. Multiple comparisons were assessed through Kruskal–Wallis test with Dunn’s multiple comparison testing and paired comparisons throughout treatment was done by the Friedman analysis of variance.

In line with the decreased lymphocyte percentages, flow cytometry studies showed a decline in the percentages of CD4+ and CD8+ T lymphocytes at time 0 (**Figures 1A, B**, respectively), with their values starting to increase following the initiation of anti-TB treatment to the levels recorded in HCo. There were no between-group differences in B-lymphocyte numbers (**Figure 1C**), but the monocyte numbers did increase in TB patients either at diagnosis or during the T6 and T9 time point evaluations (**Figure 1D**, p < 0.05 vs. HCo in all cases).

#### 3.1.1 PPARγ and PPARα expression in PBMC of TB patients

**Figure 2:**
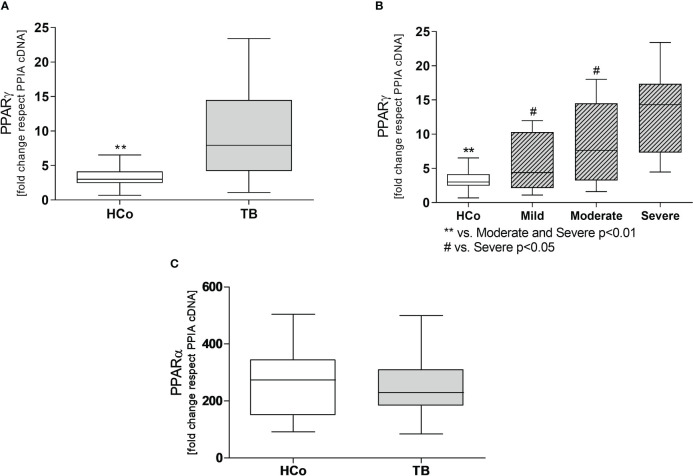
Expression levels of mRNA for PPARγ **(A)** and PPARα **(C)** in PBMC from HCo and TB patients. PPARγ mRNA expression in TB patients with different degrees of pulmonary involvement **(B)**. Boxes represent the median (line) and interquartile range, with minimal and maximum values. HCo (n = 24): healthy controls; TB (n = 39): patients with tuberculosis at the time of diagnosis. Comparisons between the two groups were made by the Mann-Whitney U test, **p<0.01. Multiple comparisons were assessed through Kruskal–Wallis test with Dunn’s multiple comparison testing **(B)**.

**Figure 3:**
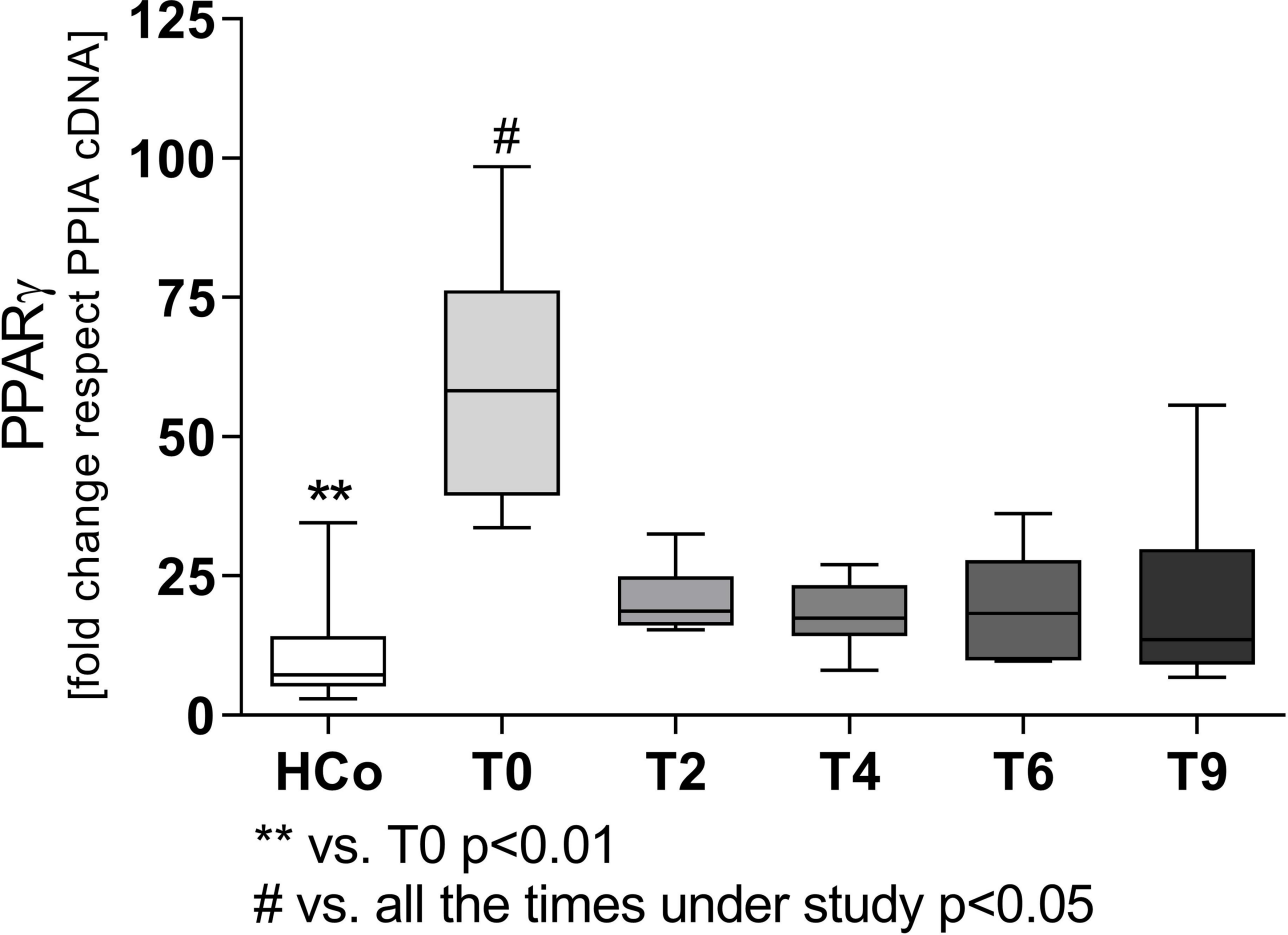
Expression levels of mRNA for PPARγ in patients with TB undergoing specific treatment. Boxes represent the median (line) and interquartile range, with minimal and maximum values. HCo (n = 24): healthy controls; T0: time at diagnosis; T2, T4, and T6: 2, 4, and 6 months following the initiation of anti-bacillary treatment; T9: 3 months following treatment completion. A total of 24 TB patients were studied at all time points. Multiple comparisons were assessed through Kruskal–Wallis test with Dunn’s multiple comparison testing, and paired comparisons during treatment were done by the Friedman analysis of variance.

Regarding the expression levels of mRNA-PPARγ in PBMC from patients with TB, values were higher at T0 compared to those recorded in HCo (**Figure 2A**), in turn, these increases were found related to the degree of lung involvement (**Figure 2B**). After two months of specific treatment, PPARγ transcripts levels, decreased to the values found in HCo (p < 0.05 vs. T0, **Figure 3**). On the other hand, mRNA-PPARα levels did not differ between TB patients and HCo (**Figure 2C**).

**Figure 4:**
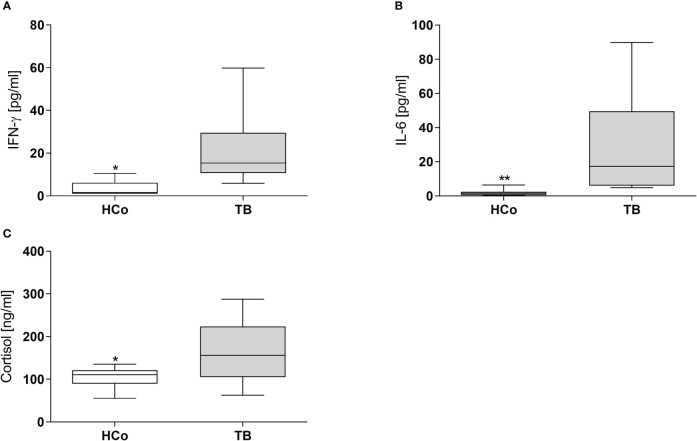
Plasma levels of IFN-γ, IL-6, and Cortisol in HCo and TB patients at the time of diagnosis. Boxes represent the median (line) and interquartile range, with minimal and maximum values. HCo (n = 24): healthy controls; TB (24): patients with tuberculosis. Comparisons between groups were made by the Mann-Whitney U test. *p<0.05; **p<0.01.

**Figure 5:**
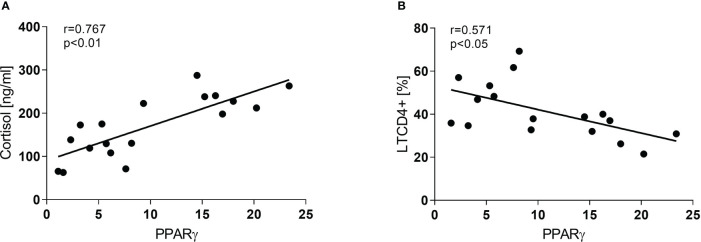
Correlation between PPARγ and Cortisol **(A)** and LTCD4+ **(B)** in TB patients at the time of diagnosis. Associations between variables were analyzed using the Spearman correlation test.

Concerning the immune-endocrine profile, newly diagnosed TB patients had increased plasma levels of IFN-γ, IL-6, and cortisol (**Figures 4A–C** respectively). The latter was positively correlated with PPARγ mRNA levels (**Figure 5A**, r = 0.767, p < 0.01). At the same time, we also found a negative correlation between PPARγ mRNA levels and LTCD4+ (**Figure 5B**, r = -0.571, p < 0.05). We have formerly demonstrated that inflammatory mediators during specific anti-TB treatment reach values similar to those of HCo, while Cortisol remains elevated throughout treatment (Díaz et al., 2017).

#### 3.1.2 PPARγ transcript expression of THP1-Mf cells stimulated with Mtbi and treated with cortisol and agonist of PPARγ

**Figure 6:**
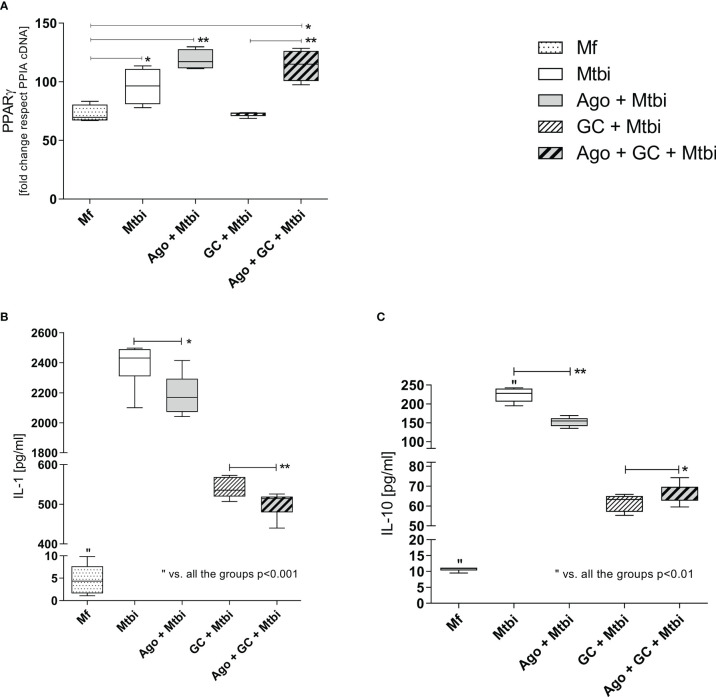
PPARγ transcript expression **(A)**, and IL-1β **(B)** and IL-10 **(C)** levels in supernatants of THP1-Mf cells stimulated with Mtbi and treated with cortisol and/or agonist of PPARγ. Boxes represent the median (line) and interquartile range, with minimal and maximum values (n=4). Mtbi: Mycobacterium tuberculosis strain H37Rv killed by γ radiation; Ago: PPARγ agonist, 15-Deoxi-Δ12,14 Prostaglandin J2 (15dPGJ2) 2μM; GC: cortisol 10-6M. Multiple comparisons were assessed through Kruskal–Wallis test with Dunn’s multiple comparison testing. *p<0.05; **p<0.01.

Following the search for the presence of PPARγ during active TB and its relationship with compounds from the immune-endocrine response we next wished to explore the potential contribution of PPARγ to the macrophage function, in terms of cytokine production, which constitutes a relevant issue during Mtb infection. As depicted in **Figure 6**, stimulation of THP1-Mf cells with Mtbi increased the expression of PPARγ (panel A) as well as the production of IL-1β and IL-10 (panels B and C, respectively). Further experiments by exposing cells to a PPARγ agonist resulted in decreased levels of both cytokines (panels B and C) together with increased amounts of PPARγ transcripts (**Figure 6A**).

The Mtbi-driven increased synthesis of IL-1β and IL-10 was no longer seen when adding cortisol to stimulated cultures (**Figures 6B, C**), without significant changes in the expression levels of mRNA-PPARγ (**Figure 6A**). In cultures undergoing Mtbi stimulation plus cortisol treatment, the addition of PPARγ agonist increased the receptor transcript expression (**Figure 6A**), whereas IL-1β and IL-10 levels appeared respectively decreased and increased (**Figures 6B, C**). There were no differences in mRNA-PPARγ levels when comparing cultures treated with agonists alone or left untreated (data not shown).

**Figure 7:**
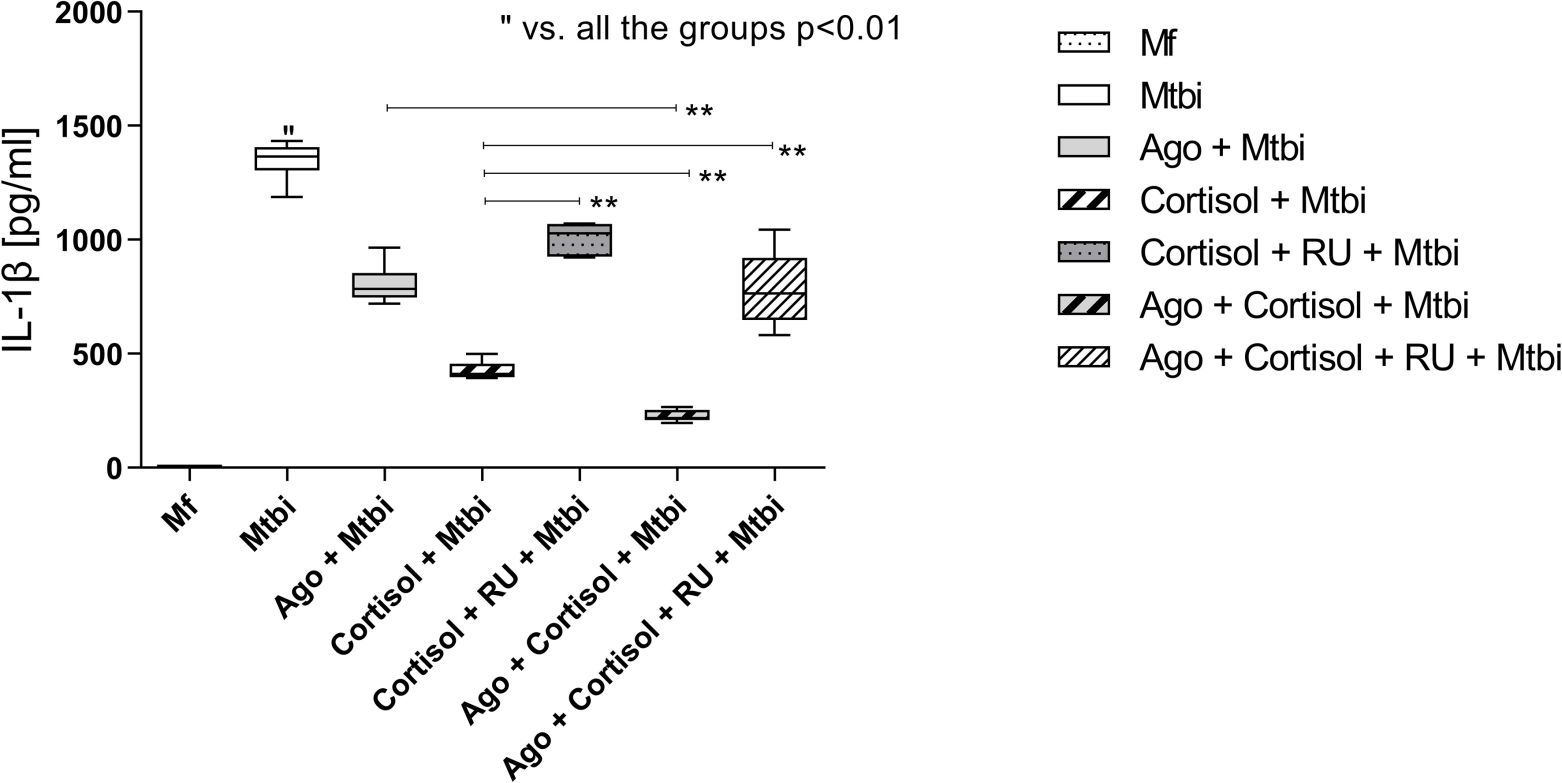
IL-1β levels in supernatants of THP1-Mf cells stimulated with Mtbi and treated with cortisol and/or agonist of PPARγ and antagonist of cortisol. Boxes represent the median (line) and interquartile range, with minimal and maximum values (n=4). Mtbi: Mycobacterium tuberculosis strain H37Rv killed by γ radiation; Ago: PPARγ agonist, 15-Deoxi-Δ12,14 Prostaglandin J2 (15dPGJ2) 2μM; RU486: glucocorticoid receptor antagonist, Mifepristone 1μM; GC: cortisol 10-6M. Multiple comparisons were assessed through Kruskal–Wallis test with Dunn’s multiple comparison testing. **p<0.01.

An additional experiment by treating cells with the cortisol receptor antagonist, RU486, showed that such treatment reversed the cortisol-reduced production of IL-1β from Mtbi-stimulated cultures (**Figure 7**). Likewise, there were no differences in the expression of PPARγ transcripts in culture counterparts exposed to the cortisol antagonist (data not shown).

## 4 Discussion

The control of an infectious process depends on the type and magnitude of the defensive response that appears beneficial during the initial phase but may become harmful if prolonged due to pathogen persistence, as is the case of TB. Because of its chronic nature, TB coexists with an immuno-endocrine-metabolic imbalance and excessive inflammatory reactions accounting for the host impairment seen during the progressive disease (Bottasso et al., 2013). In fact, at the time of diagnosis, TB patients showed a decrease in their BMI, an excessive pro-inflammatory response (Díaz et al., 2017), together with an increased NLR. The latter may be a surrogate reflecting the balance between two facets of the defensive reaction: acute and chronic inflammation (Song et al., 2021) coexisting at the same time. Another feature of the inflammatory response during TB was the increased percentage of eosinophils, at T0 and during the specific treatment, which may be related to the recruitment of these cells to exert a protective role against infection (Prakash Babu et al., 2019; Bohrer et al., 2021).

The control of the proinflammatory response requires not only intrinsic regulatory mechanisms from both innate and adaptive immune systems but also the ones extrinsic to the immune system, for instance, the activation of the hypothalamic-pituitary-adrenal axis leading to cortisol production (Kaufmann, 2001) as well as other regulators of inflammatory or immune responses like PPARs.

Within this setting, we here provide novel evidence that PBMC from newly diagnosed patients with pulmonary TB has an increased expression of PPARγ transcript, related to the degree of lung involvement, proinflammatory plasma mediators, and cortisol levels. The increased PPARγ expression coupled with elevated plasma cortisol concentrations may mirror a regulatory attempt for the substantial inflammatory response that patients show at the time of diagnosis, aimed to ameliorate the tissue damage and return to homeostasis. Without being mutually exclusive, the concomitant rise of plasma cortisol levels and PPARγ transcripts in relation to disease severity may also reflect the degree of immuno-endocrine-metabolic imbalance. Extending our former results that plasma levels of proinflammatory mediators started to decrease by the second month of specific treatment (Díaz et al., 2017), we now show that PPARγ transcripts also decay by the same time point. Cortisol levels remained elevated throughout treatment (Díaz et al., 2017), suggesting a higher role of cortisol immunomodulation during disease recovery (Díaz et al., 2017).

While the changes in the peripheral compartment may not be an accurate reflection of the response that takes place at the injury site (D’Attilio et al., 2013), they bear some relationship with the extent of lung compromise, as specific anti-TB treatment induced a significant drop of PPARγ transcript expression and in proinflammatory mediators, possibly related to the lower bacterial burden (Sabiiti et al., 2020; Osei-Wusu et al., 2021).

There is evidence that PPARγ expression following TCR stimulation of T cells negatively regulates their activation by inhibiting the nuclear factor of activated T-cells (NFAT) and subsequent IL-2 production (Clark et al., 2000; Choi and Bothwell, 2012). Yang and col. also showed that the activation of this receptor in human T cells reduced IL-2 production and proliferation (Yang et al., 2000). This may help to explain our findings about the negative correlation between PPARγ and LTCD4+ cells seen in TB patients at the time of diagnosis, wherein the former was found to be significantly increased and the latter decreased. Further studies are needed to elucidate the appropriate meaning of this association. Whether PPARγ activation is also likely to promote the biased Th2 response seen in progressive TB (Stark et al., 2021) also remains to be established.

PPARs can be expressed in a variety of tissues and cell types including Mf (Chinetti et al., 1998; Rigamonti et al., 2008). These cells are among the first ones to encounter Mtb and play an important role in regulating the immune response against this pathogen. In Mf, PPARγ would act by modifying the expression of different inflammatory genes, modulating cell differentiation and activation through the trans-repression of different transcription factors such as NF-κB, AP-1, and STATs (Ricote et al., 1999), as well as diminishing the respiratory burst (von Knethen and Brune, 2002).

The expression of PPARs can also be increased upon pathogen exposures (Penas et al., 2013). In this regard, up-regulation of PPARγ expression on Mf after infection by *Mycobacterium bovis* (BCG), Mtb, *Listeria monocytogenes*, and *Mycobacterium leprae* has been reported (Straub et al., 2002; Chan et al., 2010; Rajaram and Brooks, 2010; Mahajan et al., 2012). Almeida et al. demonstrated that infection with Mtb increases the expression and activation of PPARγ leading to an enhanced formation of lipid droplets (foamy Mf), in addition to modifying the activation profile of Mf (inhibition of proinflammatory cytokines) inducing an M2 profile (Almeida et al., 2012). Furthermore, Guerrini et al. showed that during Mtb infection, the development of Mf into foam cells involves the activation of signaling pathways leading to intracellular triglycerides accumulation, driven by TNF receptor signaling and the downstream activation of the caspase cascade and the mammalian target of rapamycin complex 1 (mTORC1) (Guerrini et al., 2018). Both mechanisms favor mycobacterial survival.

Regarding PPARγ, recognition of Mtb by Mf mannose receptor activates mitogen-activated protein kinase (MAPK)-p38-cytosolic phospholipase A2 (cPLA2), resulting in hydrolysis and release of arachidonic acid from the plasma membrane to generate prostaglandin E2 (PGE2) and cyclopentenone prostaglandins (15-d-PGJ2) (Rajaram and Brooks, 2010). These two compounds are natural ligands of PPARγ, for which they are likely to activate the receptor pathways in these cells. In line with this, present results showed that Mtb stimulation of THP1-Mf significantly increased pro and anti-inflammatory cytokines (IL-1β and IL-10) as well as PPARγ expression, whereas the activation of this receptor by a specific natural agonist diminished the expression of these cytokines in stimulated Mf at the early step of immune response (Ricote et al., 1999). Stimulated cultures treated with cortisol together with the PPARγ agonist showed the lowest levels of the proinflammatory cytokine IL-1β, suggestive of intercommunication between both immunomodulatory mechanisms. However, treatment with RU486 only reversed the inhibitory effect of GC without modifying the one exerted by PPARγ activation. This suggests that both effects may be mediated through different intracellular pathways, as reported by Yamamoto et al. in a mouse model of acute inflammation (Yamamoto et al., 2014). Although in studies employing colonic epithelial cells, cortisol was likely to control the expression of PPARγ (Bouguen et al., 2014).

In non-infectious chronic diseases, i.e., metabolic syndrome or diabetes, the use of PPARγ agonists, like TZDs (including rosiglitazone and pioglitazone), showed some effectiveness in parallel to side effects such as increased weight gain, fluid retention, bone loss, congestive heart failure, etc. (50), which led to its discontinuation as was the case of rosiglitazone (51). This stimulated the search for new antagonists exempted from such adverse effects. Our results along with the relevance of lipid metabolism in the pathogenesis of TB and Mtb survival, as well as the fact that PPARγ deletion in pulmonary Mfs improved the course of murine tuberculosis (52), provide no evidence to think about the potential use of a PPARγ agonist in TB.

In getting a better understanding of the role of PPARγ in the host-mycobacteria relationship, our study provides the first evaluation of the expression of PPARγ in PBMC from TB patients according to the extent of lung damage and specific treatment. Results provide a stimulating background for further analysis of the interrelation between PPARs and the immune-endocrine framework of this particular disease.

## Data availability statement

The raw data supporting the conclusions of this article will be made available by the authors, without undue reservation.

## Ethics statement

The studies involving human participants were reviewed and approved by the study protocol by the Ethical Committee of the Faculty of Medical Science, National University of Rosario (Resolution n° 44405/0005) and the Centenario Hospital of Rosario (Resolution n° 528). The patients/participants provided their written informed consent to participate in this study.

## Author contributions

All authors have contributed to this project. All authors have read and agreed to the published version of the manuscript.
